# Supplementary material for: Complexome profiling of the Chlamydomonas psb28 mutant reveals TEF5 as an early PSII assembly factor
Source: Plant Cell. 2025 Mar 17;37(6):koaf055. doi: 10.1093/plcell/koaf055 (PMC12164590; doi:10.1093/plcell/koaf055)
Supplement: koaf055_Supplementary_Data [file koaf055_supplementary_data.zip › psb28_interactive.zip!/plots/intensity/qualityPlots/peptides/VDAC2_Cre05.g241950.t1.2.html]

### Cre05.g241950.t1.2

Trivial name: VDAC2  
SeqCoverage [median]: 65.60  
Mass: 28.55 kDa  
  
 

| Sequence | MissedCleavage | Mass [Da] | wtIntensity | muIntensity | Score |
| --- | --- | --- | --- | --- | --- |
| ADKVEASLK | 1 | 959.53 | 1415.22 | 0.00 | 140.45 |
| GLLGGDKPTGTFSFDPK | 1 | 1735.88 | 15445.05 | 75513.29 | 101.53 |
| KLATSDTTFALAYAR | 1 | 1627.86 | 26111.43 | 57835.70 | 132.08 |
| KYSVDVALSPDNK | 1 | 1434.74 | 2063.54 | 6236.38 | 109.24 |
| LATSDTTFALAYAR | 0 | 1499.76 | 6935.86 | 25056.94 | 227.36 |
| LDGSGALSALYETK | 0 | 1423.72 | 3176.08 | 9309.39 | 146.36 |
| LIYSHNLTSTSTVGAEVTR | 0 | 2048.05 | 5573.62 | 31922.51 | 131.67 |
| LKLDGSGALSALYETK | 1 | 1664.90 | 3023.86 | 22209.04 | 81.43 |
| LSVSSTTASGVALTATTVQK | 0 | 1921.04 | 832.44 | 1346.51 | 47.09 |
| LTLDYSMPYLALK | 0 | 1526.81 | 23822.72 | 38142.72 | 206.73 |
| LTTSAVLPDPATAK | 0 | 1383.76 | 44925.23 | 103505.09 | 196.11 |
| PVVSFGDIGK | 0 | 1017.55 | 89772.42 | 184342.66 | 200.54 |
| PVVSFGDIGKAAK | 1 | 1287.72 | 1361.40 | 4040.18 | 95.42 |
| VTATASINDVAPGIK | 0 | 1455.79 | 11742.39 | 29632.95 | 228.73 |
| VTGSLQLQATDLSK | 0 | 1459.79 | 938.59 | 3271.22 | 238.20 |
| VTGSLQLQATDLSKPVK | 1 | 1784.00 | 19875.25 | 88418.58 | 166.48 |
| YSVDVALSPDNK | 0 | 1306.64 | 443.03 | 4328.63 | 143.40 |
|  |  |  | 257458.1 | 685111.8 |  |
